# Primary Involvement of Pharynx and Peyer's Patch in Inhalational and Intestinal Anthrax

**DOI:** 10.1371/journal.ppat.0030076

**Published:** 2007-06-01

**Authors:** Ian J Glomski, Alejandro Piris-Gimenez, Michel Huerre, Michèle Mock, Pierre L Goossens

**Affiliations:** 1 Unité des Toxines et Pathogénie Bactérienne, Institut Pasteur, Paris, France; 2 CNRS, URA 2172, Paris, France; 3 Unité de Recherche et d'Expertise en Histotechnologie et Pathologie, Institut Pasteur, Paris, France; Salk Institute for Biological Studies, United States of America

## Abstract

Bacillus anthracis causes three forms of anthrax: inhalational, gastrointestinal, and cutaneous. Anthrax is characterized by both toxemia, which is caused by secretion of immunomodulating toxins (lethal toxin and edema toxin), and septicemia, which is associated with bacterial encapsulation. Here we report that, contrary to the current view of B. anthracis pathogenesis, B. anthracis spores germinate and establish infections at the initial site of inoculation in both inhalational and cutaneous infections without needing to be transported to draining lymph nodes, and that inhaled spores establish initial infection in nasal-associated lymphoid tissues. Furthermore, we found that Peyer's patches in the mouse intestine are the primary site of bacterial growth after intragastric inoculation, thus establishing an animal model of gastrointestinal anthrax. All routes of infection progressed to the draining lymph nodes, spleen, lungs, and ultimately the blood. These discoveries were made possible through the development of a novel dynamic mouse model of B. anthracis infection using bioluminescent non-toxinogenic capsulated bacteria that can be visualized within the mouse in real-time, and demonstrate the value of in vivo imaging in the analysis of B. anthracis infection. Our data imply that previously unrecognized portals of bacterial entry demand more intensive investigation, and will significantly transform the current perception of inhalational, gastrointestinal, and cutaneous B. anthracis pathogenesis.

## Author Summary

Anthrax is caused by *Bacillus anthracis,* a bacterial pathogen that forms spores, dormant bacteria that are highly resistant to destruction. Infections initiate from the introduction of spores into airways or damaged skin, or from the consumption of contaminated food. Within the host, spores germinate, then bacteria secrete toxins that cripple the immune response and sheath themselves in a capsule that prevents them from being phagocytosed. We strove to determine in real space and time where and when spores introduced by these three routes of infection germinate and how bacteria subsequently disseminate in a mouse model. This was achieved through the development of light-emitting B. anthracis that could be tracked inside a living mouse. Contrary to current models, our studies indicated that spores germinated in situ in the skin, the intestines, and the nasal passages without needing to be transported to lymph nodes. Furthermore, bacteria disseminate from initial sites of infection in a similar fashion, first to the draining lymph nodes, then the spleen, and finally the lungs and blood. These findings imply that spore interactions with local sites of entry are critical in the development of systemic disease and that disruption of these interactions may offer new methods of anthrax prevention.

## Introduction

Bacillus anthracis is a sporulating Gram-positive bacterium that causes the disease anthrax. The three forms of anthrax reflect the route by which the infection is initiated: cutaneous, gastrointestinal, and inhalational [1,2]. Anthrax is characterized by both toxemia, which is caused by secretion of two toxins, and septicemia, which is associated with bacterial encapsulation. The capsule consists of poly-γ-d-glutamic acid attached to the peptidoglycan of the cell wall and inhibits phagocytosis, functions as a non-immunogenic surface, and is vital for full virulence [3–5]. The toxins, lethal toxin and edema toxin, modulate host immune responses and at high doses can cause death [6,7], but elimination of toxin production does not alter virulence in a mouse model of B. anthracis infection [8,9].

Surprisingly, relatively little is known about how spores enter the host or how the dynamics of infection are affected by the route of infection, and no animal model exists for gastrointestinal anthrax at this time. Cutaneous anthrax, the most common yet least lethal form of anthrax in humans, is believed to initiate through abrasions in the skin [10]. Gastrointestinal anthrax is generally considered to be the primary route of infection of livestock, can occur in humans, and is caused through the ingestion of contaminated food [2,11], yet the means by which B. anthracis crosses membrane barriers to establish infection remains unknown. The current model of inhalational anthrax consists of uptake of spores by alveolar macrophage, then transport of these spores to the draining mediastinal lymph nodes, where the spores germinate and establish infection within the lymphatics to ultimately disseminate systemically [10,12,13].

To identify the portals of initial entry and growth and to better define the differences associated with these three forms of B. anthracis infection, a model using in vivo bioluminescent imaging (BLI) was developed. BLI consists of detecting photons emitted from a cell expressing luciferase—in this case, a bacterium—within the body of a host animal [14]. BLI analysis of B. anthracis infection provides the advantages of tracking a dynamic infection in a dynamic fashion, monitoring the entire mouse (allowing the discovery of as yet-unknown locations of bacterial growth), and detecting subclinical infections. Thus, BLI permits a kinetic and global view of bacterial dissemination that allows the synthesis of an integrated infection model from inoculation to death.

We found that, contrary to the current view of B. anthracis pathogenesis, B. anthracis spores germinate and establish infections at the initial site of inoculation in both inhalational and cutaneous infections without needing to be transported to draining lymph nodes. Furthermore, we found that Peyer's patches in the mouse intestine are the primary site of bacterial growth after intragastric inoculation, establishing, to our knowledge, the first animal model of gastrointestinal anthrax. Our data imply that previously unrecognized portals of bacterial entry demand more intensive investigation.

## Materials and Methods

### Bacterial Strains, Growth Conditions, and Reagents

All experiments were performed with a bioluminescent derivative of B. anthracis strain 9602P (described previously in [8]), which is a derivative of the highly virulent natural human isolate 9602 [15], but does not produce the protective antigen component of the toxins. Bacteria were grown on brain–heart infusion (BHI) agar (Difco, http://www.bd.com/ds) unless otherwise noted. Spores were produced and purified on Radioselectan (Renografin 76%; Schering, http://www.schering.de) using previously described methods and stored in sterile deionized water [16].

### Construction and Verification of Bioluminescent B. anthracis

The *luxABCDE* operon was PCR amplified from pXEN5 (pAUL-A Tn*4001 luxABCDE Km^r^* from the Xenogen Corporation, http://www.xenogen.com) [14] using the following oligonucleotides: LuxAF1BamXho-CGCGGATCCTCGAGCAGATGAAGCAAGAGGAGGACTC, which contains a BamHI followed by an XhoI site (underlined); LuxER1Bam- GGCGGATCCGTCGACTTAACTATCAAACGCTTCGG, which contains a BamHI site. This PCR product was ligated with the pGEM-T Easy vector kit (Promega, http://www.promega.com) and transformed into Escherichia coli strain TG1 (pIG3–40). The pIG3–40 plasmid was subsequently purified, and the *luxABCDE* insert was removed via BamHI (New England Biolabs, http://www.neb.com) digestion and ligated into plasmid pAT113 [16], a conjugative shuttle vector that has no Gram-positive origin of replication (pIG4–2). The *pag* promoter, consisting of 1 kb of sequence upstream of the protective antigen coding sequence, was PCR amplified from B. anthracis DNA using the following oligonucleotides: PPagF1EcoR1-CGAGAATTCGATGAAAATGGTAATATAGCG, which contains an EcoRI site, and PPagR1Xho- AGGGCTCGAGGTATAAAATTAAATTTATATTATATTG, which contains an XhoI site. This PCR product was cut with EcoRI and XhoI, ligated into pIG4–2, cut by equivalent enzymes, and transformed into TG1 (pIG6–19). Subsequently, pIG6–19 was introduced into B. anthracis 9602P by heterogramic conjugation using previously described methods and the conjugative E. coli strain HB101 (pRK212.1) [16], and selected on BHI plus 5 μg/ml erythromycin, yielding strain BIG19. Insertion into the upstream sequence of *pag* was verified by both PCR and the production of light when grown on capsulation plates in 5% CO_2_ at 37 °C [17]. The stability of the *lux* integration was assessed by diluting cultures 1:1,000 in R-media with 0.6% sodium bicarbonate (R-Bic) [18] at 37 °C and 5% CO_2_ two times daily for 1 wk, then detecting the presence of the *lux* insertion by erythromycin resistance and luminescence. All clones contained the insert after 1 wk of passage.

Determination of bioluminescence in comparison to growth phase was performed by growing BIG19 in BHI plus 0.5% glycerol media overnight at 37 °C with agitation. Subsequently, this culture was diluted into R-Bic to an optical density (OD) of 0.05, and then incubated at 37 °C in 5% CO_2_ with agitation. At the indicated times, samples were removed and imaged for luminescence in a 96-well plate (Nunc, http://www.nuncbrand.com) with the IVIS 100 system as described below, and the OD600 was measured with a spectrophotometer (Ultrospec 3300 *pro;* Amersham Biosciences, http://www.gelifesciences.com).

### Infection and Imaging

Six- to 10-week-old BALB/cJ mice (Charles River Laboratories, http://www.criver.com) were maintained under specific pathogen-free conditions at the Pasteur Institute in compliance with European animal welfare regulations. Cutaneous infections were performed under light anesthesia by injecting 10 μl of spore suspension in PBS into the dermis of the right ear with a 0.5-ml insulin syringe (Becton Dickinson, http://www.bd.com) [19]. For the intravenous infection experiments, capsulated bacteria were prepared by germinating spores in BHI for 5 min at room temperature, centrifuging, and incubating in R-Bic induction media at 37 °C in 5% CO_2_ with agitation for 1.5 or 5 h. The length of bacteria in each sample was quantified by binding formalin-fixed bacteria to polylysine-coated glass cover slips, acquiring digital microscopic images, and then measuring the length of the bacteria in each image. Injection cultures were washed with RPMI-Glutamax (Invitrogen, http://www.invitrogen.com), and 1.4 × 10^7^ and 2.0 × 10^6^ colony-forming units (CFU) of the 1.5- and 5-h cultures, respectively, were injected. Seven times less CFU of the 5-h culture was injected to account for the fact that the 5-h bacteria were approximately seven times longer than the 1.5-h bacteria, and thus CFU were not an accurate measure of bacterial mass. Spores in 100 μl of PBS were inoculated intragastrically by inserting a 5-cm length of polyethylene catheter that was attached to a needle and syringe down the esophagus and into the stomach of lightly anesthetized mice, changing the catheter for each mouse. Alternately, 18G animal feeding needles (Popper and Sons, http://www.popperandsons.com) were used for intragastric inoculation. Aerosol infections were performed by delivering spores for 20 min using a Raindrop nebulizer (Tyco, http://www.tyco.com) designed to produce 1-μm droplets of a solution containing 1 × 10^8^ spores/ml water in an all-glass, nose-only aerosol chamber [20]. An average 3.8 ± 0.3 log_10_ (mean ± standard deviation [SD]) spores were found in the lungs 2 h post-infection. Intranasal inoculation was performed in lightly anesthetized mice by introducing 1 × 10^5^ spores in 20 μl of PBS upon inhalation into the right nostril. An average of 4.5 ± 0.1 log_10_ (mean ± SD) spores were found in the lungs 2 h post-infection. Intratracheal inoculation of 1 × 10^5^ spores was performed using plastic tubing as previously described [21]. An average of 3.8 ± 0.2 log_10_ (mean ± SD) spores were found in the lungs 2 h post-infection. For CFU determination, organs were immediately placed in ice-cold saline solution and then homogenized in a chilled glass tube, and dilutions were plated on BHI plates. Differentiating spores from vegetative bacteria was determined after heat treatment of 65 °C for 30 min to kill the vegetative bacteria. Lymph node location and nomenclature were based on previously defined standards [22]. Lungs were isolated by cutting below the tracheobronchal bifurcation where the bronchus enters the lobes of the lung.

Images were acquired using an IVIS 100 system (Xenogen) according to instructions from the manufacturer. Analysis and acquisition was performed using Living Image 2.5 software (Xenogen). Unless otherwise noted, mice were anesthetized using a constant flow of 2.5% isofluorane mixed with oxygen using an XGI-8 Gas Anesthesia System (Xenogen), which allows control of the duration of anesthesia. Images were acquired with a binning of 16. Luminescent signals from the exterior of mice or cultures in plates were acquired for 1 min, whereas luminescence of internal organs during dissection was integrated for 10 s. All other photographic parameters were held constant. Quantifying photons per second emitted by each organ was performed by defining regions of interest corresponding to the organ of interest, while subtracting the background as defined by a non-infected animal in the same photograph.

### Histology

Tissues were removed from mice with luminescence in the organ of interest and immediately placed in 4% buffered formalin (Labonord, Templemars, France) for standard microscopic analysis. Skulls of mice were decalcified in a solution of 4% buffered formalin and 10% trichloroacetic acid for approximately 2 mo for analysis of the nasal passages. Serial 5-μm sections were stained with hematoxylin and eosin and/or Gram [23]. Samples were analyzed by two independent observers, one of whom was a trained pathologist.

### Statistical Analysis

**Figure 1:**
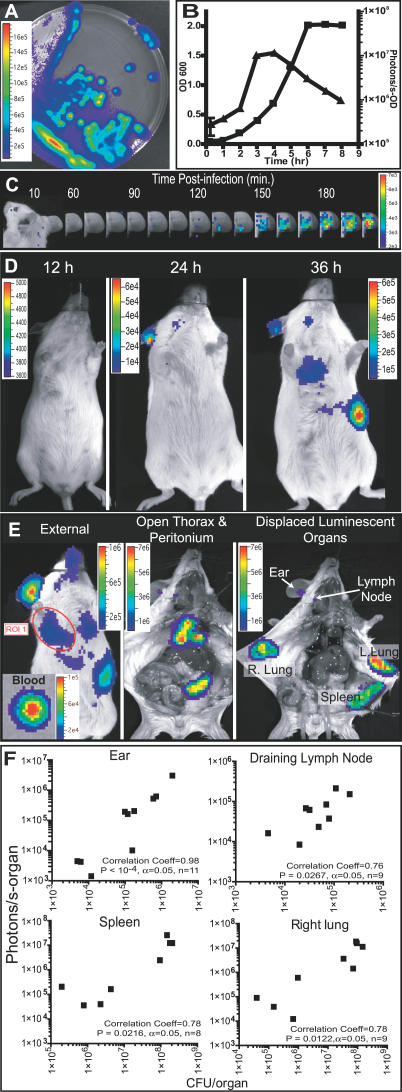
Bioluminescent B. anthracis Allow Efficient Spatiotemporal Determination of In Vivo Bacterial Location and Quantification in a Cutaneous Model of Infection In all figures, black and white photographs are overlaid with false color representation of the photons s^−1^ cm^−2^ sr^−1^; the false color scale is incorporated into each image or group of images. (A and B) B. anthracis carrying *luxABCDE* (strain BIG19) are luminescent on solid (A) or liquid (B) capsulation medium. (B) Bioluminescence is maximal during mid-exponential phase (squares, optical density; triangles, photons s^−1^ OD^−1^). (C) Monitoring of light production at 10-min intervals after cutaneous infection (1 × 10^4^ spores). Image series is representative of four mice. (D) Image series of a cutaneous infection in the ear (5 × 10^2^ spores) acquired at the indicated times (representative of 15 mice). (E) Dissection at 52 h post-infection. Left: unopened mouse, with an example of a measurement region of interest (ROI 1) encompassing the right lung, and an inset of a drop of blood. Center: open peritoneum and thorax. Right: displacement of organs to identify the sources of luminescence. (F) Correlation between external quantification of the photons emitted from a defined region of interest encompassing the organ and CFU counts for the same organs; each dot represents one organ.

Statistical analysis and graphing was performed using GraphPad Prism 4 software (http://www.graphpad.com). The Pearson correlation coefficients (*r*) were used to determine the direction and magnitude of covariation between CFU and photons/s-organ, since each X and Y value were independent measures and are assumed to follow a Gaussian distribution. The two-tailed *p*-values, α, and *n* are indicated on each graph in Figure 1F.

## Results

A capsulated non-toxinogenic strain of B. anthracis was chosen for study because the capsule is vital for virulence and is associated with dissemination [3–5], while elimination of toxin production does not alter virulence in mouse models of infection [8,9]. Bioluminescent B. anthracis were constructed by integrating the *luxABCDE* genes from Photorhabdus luminescens [14] under the control of the *pag* promoter, the gene for protective antigen, a toxin component that is highly induced under in vivo conditions [18]. These bacteria were strongly luminescent (Figure 1A), whereas dormant spores were not (unpublished data), reflecting the necessity of bacterial metabolic activity for light production. Maximal luminescence was observed during mid-log phase growth and decreased in late-log and stationary phase (Figure 1B). Spores derived from these bioluminescent bacteria were used in subsequent murine bacterial dissemination models.

Cutaneous infection is the most common form of anthrax in humans and is often limited to the site of initial infection [1]. A cutaneous ear infection model [19] allowed efficient enumeration of CFU and high sensitivity. Luminescence at the site of injection was detected within 120 min (Figure 1C). This shows that spores rapidly germinate at the site of injection and quickly become metabolically active, and that the *pag* promoter is induced; these results are similar to those obtained in tissue culture models [24,25], but unlike previous studies that suggested that germination occurs in the draining lymph nodes [7,10]. Within 24 h of cutaneous injection of 500 spores, bioluminescence was detected in both the ear and the superficial parotid lymph node draining the ear, and was also visible in the spleen and right lung 12 h later (Figure 1D). Dissection confirmed that the superficial parotid lymph node, spleen, both lungs, and the blood were luminescent (Figure 1E). The left lung, though luminescent, was more difficult to detect from the exterior because the heart partially blocked the exit of photons. No other organs harbored luminescent B. anthracis.

Differences in pigmentation, organ orientation, and tissue depth affect how light exits the body, necessitating determination of the relationship between luminescence and quantity of bacteria for each organ of interest. Luminescence correlated with CFU in the ear, superficial parotid lymph node, spleen, and right lung (Figure 1F). We analyzed only the right lung because the heart blocked a large portion of the left lung's emitted light. The minimum detectable quantity of bacteria in each organ was as follows: ear, 3 × 10^3^; superficial parotid lymph node, 4 × 10^3^; spleen, 2 × 10^5^; and right lung, 2 × 10^4^. Thus, an increase in luminescence at a particular location can be interpreted as an increase in the bacterial load.

**Figure 2:**
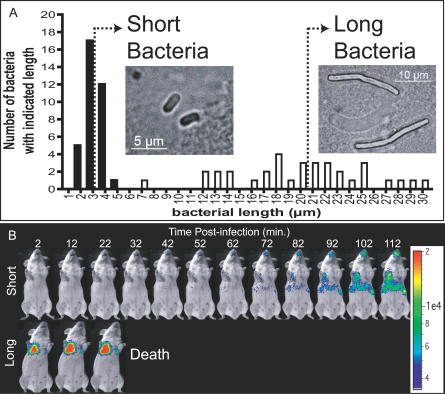
Bacterial Growth Phase Affects Hemotagenous Dissemination (A and B) Luminescent capsulated bacteria grown for 1.5 or 5 h in R-Bic medium, averaging either 3.2 ± 0.7 μm (solid bars, “Short Bacteria”) or 20.0 ± 5.5 μm (empty bars, “Long Bacteria”) in length (mean ± SD), respectively, were used to infect the mice in (B). The dotted lines indicate the median bacilli length for each bacterial population. Inset photographs depict phase-contrast microscopic images of short and long bacteria with the corresponding scale. (B) Mice infected intravenously with either short or long capsulated luminescent bacteria (see Materials and Methods), represented in (A), were analyzed as in Figure 1, at the indicated times. The scale at the right applies to all images of mice.

Lung luminescence late in infection was unexpected from cutaneous infections. This may indicate that bacteria growing in distal sites enter the blood and are trapped in the lungs [5], which is the first fine capillary bed encountered upon the return of blood from the periphery. Bacterial entry into the blood was simulated by intravenous injection. Luminescent capsulated bacteria grown 5 h in inductive media, averaging 20.0 ± 5.5 μm (mean ± SD) in length and injected intravenously, were detected solely in the lungs from 2 min post-injection until death (Figure 2A and 2B), and killed the mice within 40 min. Bioluminescence was not detected in any other organs, suggesting that a majority of the bacteria were immediately trapped in the lungs. In contrast, light emitted by bacteria grown 1.5 h, averaging 3.2 ± 0.7 μm in length, was initially below the detection threshold. After 1 h, bioluminescence was detectable in the spleen, liver, and lungs. These data suggest that bacteria from the 1.5-h culture were initially widely distributed throughout the mouse and were eventually localized within the spleen, liver, and lungs. This implies that the size of bacteria entering the blood circulation and/or growth phase influences the dissemination of bacteria to the lungs and other organs.

**Figure 3:**
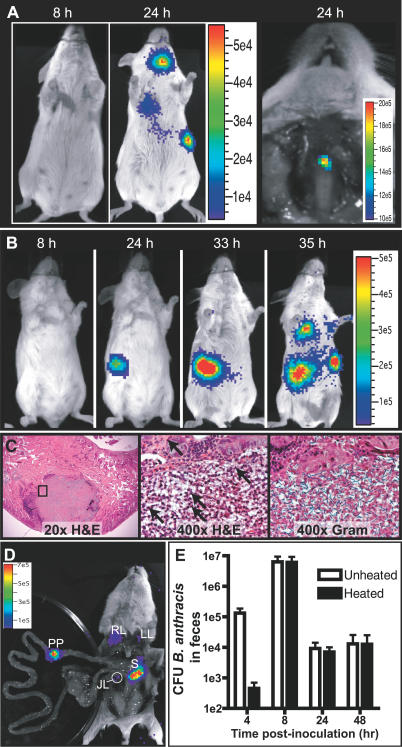
Initial Involvement of Peyer's Patches or Oropharynx in Gastrointestinal B. anthracis Infection (A–E) BIG19 (1 × 10^8^) spores were inoculated intragastrically using a rigid feeding needle (A) or a flexible plastic tube (B–E), yielding distinctly different patterns of infection. (A) Left: image series at the indicated times. Right: dissection of the anterior of the neck of the same mouse, displaying luminescence in the laryngopharynx. (B) Infection of the lower digestive tract at the indicated times. Image series is representative of 23 mice. (C) Tissue sections of an infected Peyer's patch, stained with hematoxylin and eosin (H&E) (left and middle) and Gram (right) at low and high magnification (location indicated by the black box). The arrows in the middle image point to numerous neutrophils infiltrating the Peyer's patch. (D) Dissection of the mouse in (B) to confirm sources of luminescence. JL, jejunal lymph node; LL, left lung; PP, Peyer's patch; RL, right lung; S, spleen. (E) Total CFU of B. anthracis (Unheated) or spores only (Heated) were enumerated at the indicated times from the feces of mice inoculated intragastrically (mean ± standard error of the mean, *n* = 4).

Anthrax is associated with polygastric ruminant livestock, and natural gastrointestinal infection occurs when food contaminated with B. anthracis spores is ingested [1]. Likewise, gastrointestinal anthrax occurs in monogastric hosts, such as humans [2,15], yet there are no published animal models of gastrointestinal anthrax. Intragastric inoculation of spores in the monogastric mouse using a rigid feeding needle caused infection of the laryngopharynx (Figure 3A), suggesting that abrasions in the upper alimentary tract acted as portals of infection. Intragastric inoculation with flexible plastic tubing caused true gastrointestinal infection that initiated as a defined point of luminescence in the abdomen from approximately 24- to 33-h post-inoculation that suddenly progressed systemically (Figure 3B). Histological examination of the initial site of infection showed that bacterial growth occurred in the Peyer's patch (Figure 3C and 3D), and could occur in Peyer's patches throughout the small intestine (unpublished data). Very little of the original dome architecture was maintained, with only a few lymphocytes at the periphery. Bacterial rods, numerous polymorphonuclear cells, necrosis, and hemorrhage were evident (Figures 3C and S1). Similar lesions were observed in the spleen and lung (Figure S1). Interestingly, vegetative bacteria could be isolated from the feces 4 h post-inoculation (Figure 3E), but only spores were found 8 h post-inoculation. These data suggest that spores germinate in the gastrointestinal tract, vegetative bacteria transiently colonize the lumen, and spores are continuously shed for up to 48 h post-inoculation after a majority of the initial inoculum has been excreted. As in the cutaneous model, the ensuing systemic infection spread first to the draining jejunal lymph nodes, then the spleen, and finally the lungs (Figure 3B–3D).

**Figure 4:**
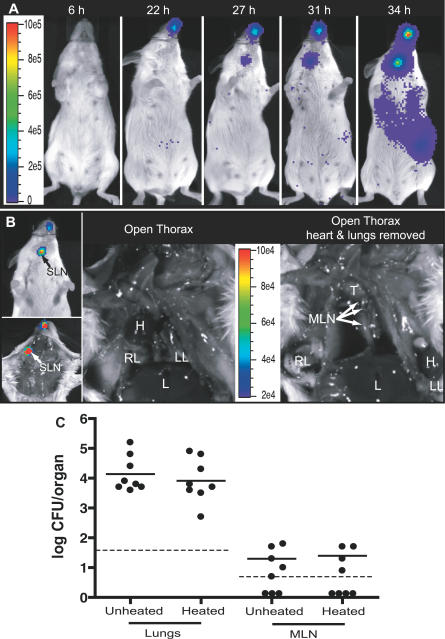
Aerosolized Spores Cause Nasopharyngeal Infection without Growth in Mediastinal Lymph Nodes (A) Image series of a mouse infected by aerosolized BIG19 spores. Images are representative of eight mice. (B) Dissection of a mouse (bottom left) early in infection (28 h) with the corresponding external image (top left) showing bioluminescence in the nasal cavities and mandibular lymph node, the open thorax (center), and with the heart and lungs displaced (right). Arrows point to the mediastinal lymph nodes (MLN) that do not display luminescence. H, heart; L, liver; LL, left lung; RL, right lung; SLN, submandibular lymph nodes; T, thymus. (C) Total CFU of B. anthracis (Unheated) or spores only (Heated) were enumerated in the lungs and tracheobronchal mediastinal lymph nodes (MLN) when luminescence was detected in the nasopharynx. Each circle represents the CFU derived from one mouse. Solid lines indicate the log-mean. Dotted lines represent the detection limits for the different organs. There was no significant difference between unheated or heated samples from the lungs (*p* = 0.62) and mediastinal lymph nodes (*p* = 0.90) (Student's *t*-test). Data are derived from two independent experiments.

**Figure 5:**
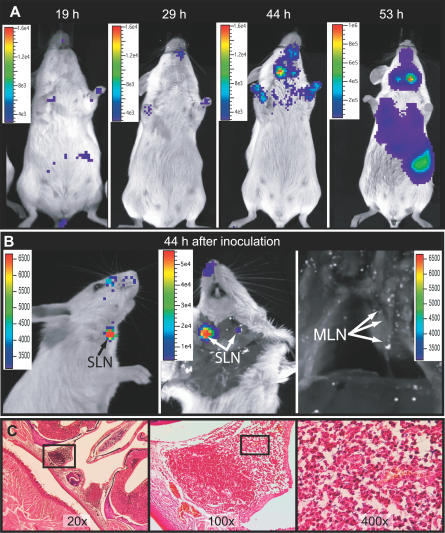
Bacterial Growth Occurs in NALTs after Intranasal Inoculation (A) Image series of a mouse infected by intranasal inoculation of 1 × 10^5^ spores at the indicated times. Image series is representative of 12 mice. (B) Images of a mouse infected for 44 h. Left: external right side. Center: opened anterior of the neck. Right: thorax with lungs and heart removed. Arrows point to the mediastinal lymph nodes (MLN) that do not display luminescence. SLN, submandibular lymph nodes. (C) Gram-stained transverse tissue sections of a bioluminescent nasal cavity 44 h post-infection. Left: 20× image of the nasal cavity. Center and right: 100× and 400× images, respectively, corresponding to the location indicated by the black boxes in the 20× and 100× images, respectively. Infected NALT displayed breakdown of the lymphoid tissue structure, infiltration of numerous polymorphonuclear cells, several areas of light hemorrhage, and numerous Gram-positive bacterial rods, and also an undamaged epithelial layer.

**Figure 6:**
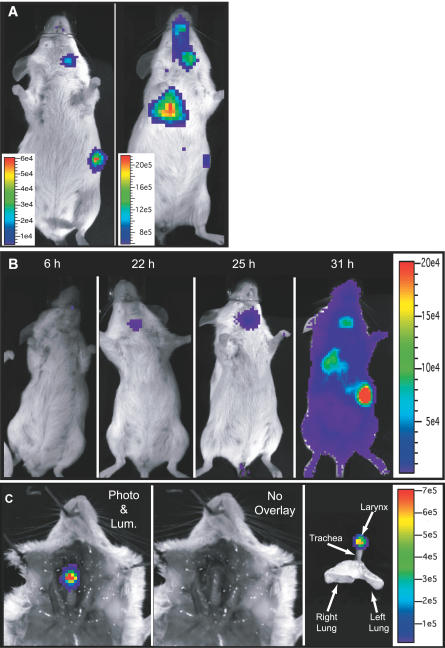
Inoculation by Intratracheal Intubation Leads to Two Different Patterns of Infection (A) Images of two representative mice infected 46 (left) and 52 h (right) after intratracheal inoculation of 1 × 10^5^ BIG19 spores that developed infection in the oropharynx. Luminescence is detected in the oropharynx, spleen, and lung. (B) Time-lapse image series of a mouse with infection of the larynx. Images are representative of 18 intratracheally infected mice. (C) Dissection of a mouse with laryngeal infection, with (left) or without (center) the luminescence overlay. Right: an image of the respiratory tract *ex vivo*.

Inhalational anthrax has a high mortality rate and is the form of disease that is targeted when spores are weaponized [26]. Dissemination kinetics of aerosol, intranasal, and intratracheal inoculation of spores were studied. Mice infected with aerosolized spores developed an infection in the nasal cavity by 22 h post-infection, followed by the mandibular lymph nodes 5 h later (Figure 4A and 4B). When bioluminescence was first visible in the nasal cavity, it was not found in the lungs and mediastinal lymph nodes (Figures 4B and 5B), despite the effective delivery of spores into the lungs (see Materials and Methods) and the presence of a small quantity of heat-resistant spores in the tracheobronchal mediastinal lymph nodes (Figure 4C). Intranasal inoculation of spores also caused infection of the nasal cavity by 29 h post-infection, followed by the mandibular lymph nodes 15 h later (Figure 5A and 5B). Histologic analysis revealed that bacteria were growing in nasal-associated lymphoid tissues (NALTs) when bioluminescence was first detected in the nasopharynx (Figure 5C). Inoculation of spores by intubation of the trachea caused two distinctly different patterns of infection. In the first form, the primary site of early luminescence was the mandibular lymph nodes (Figure 6A), likely representing initiation of infection by spores that were expelled from the lung by coughing and/or the ciliary escalator [27]. The second form of infection initiated in the larynx within 22 h post-infection and progressed to systemic disease within 9 h (Figure 6B and 6C). Intubation of the trachea (via the larynx) may cause abrasions that allowed direct mucosal infection, as previously noted [12], and which we also observed in the gastrointestinal model (Figure 3A). As in the cutaneous and intragastric models, the bioluminescence in all inhalational models progressed from the site of initial infection to systemic distribution (Figures 4A and 5A).

## Discussion

Real-time analysis of capsulated B. anthracis infection has allowed the synthesis of a coherent model of murine infection from initial infection to death that modifies the current paradigm of B. anthracis dissemination; primary bacterial growth is limited to locations associated with the inoculation site, followed by dissemination to the draining lymph nodes, then the spleen, and ultimately the lungs and blood. In agreement with J. M. Ross's seminal study of inhalational infection of guinea pigs [12], our data indicated no bacterial growth within the lungs at early stages of infection in inhalation models. These results, along with our observation that intravenous bacteria rapidly associate with the lungs, support the hypothesis that vegetative bacteria arrive in the lungs via the blood [5,28], rather than in situ growth from spores.

Unexpectedly, our results showed that metabolically active bacteria were found solely in the nasopharynx and its associated lymph nodes early in infection, which is contrary to previous studies that suggested that bacteria multiply primarily in the mediastinal lymph nodes at the initiation of infection [12,13]. These previous studies note that there were incisions or trauma to the airways caused by experimental technique, which makes interpretation of early events in the infection difficult, as we found that abrasions were preferential portals of infection (Figures 3A, 6B, and 6C) and may allow bacterial access to the lymph nodes via direct transport to the lymphatics across the damaged epithelial barrier. Indeed, in Ross's study, spores in transit to the regional lymph node are only observed in infections initiated through intratracheal intubation, a technique that she noted to cause tracheal injury, and were not observed in transit when a non-invasive spore aerosol was used [12]. BLI analysis of B. anthracis infection provided the advantage of differentiating these unintended sites of infection from those infections initiated in targeted tissues.

Furthermore, other studies that support mediastinal growth have derived conclusions from autopsy of animals after death caused by the infection [28,29], which could confound interpretation, since we observed a sudden increase in bacteria in the lungs late in infection. The bacteria found in the mediastinal lymph nodes late in infection or at death could otherwise be explained as originating from the population of bacteria that spread to the lungs hematogenously. Regardless of the method of spore delivery (cutaneous, gastrointestinal, intravenous, or by inhalation) we found that the lung eventually contained large numbers of bacteria that may subsequently drain into the mediastinal lymph nodes. Supporting this interpretation, Zaucha et al. noted significant mediastinal lymph node involvement in rabbits infected subcutaneously [28]. Thus, our investigation also implies that the timing of when tissue samples are taken is vital, indicating an additional advantage of using dynamic models of infection such as BLI.

Our findings demonstrate that a severe infection with highly metabolically active bacteria develops in the nasopharynx of mice at a time in the infection when low to undetectable quantities of spores are in the mediastinal lymph nodes. Thus, it is reasonable to question the relevance of the bacteria found in the mediastinal lymph nodes to the subsequent disseminated disease when at an earlier time-point a much larger infection is occurring in the nasopharynx. Moreover, large quantities of spores can persist in the lungs for long periods without causing disease ([30]; unpublished data), also bringing into question the relative efficacy of initiation of infection from lung tissues. Additionally, we observed bacteria growing within the NALT, which may indicate that bacteria can directly invade tissues of the nasopharynx without needing to be transported by alveolar macrophages to the lymphatics, as previously postulated [31]. Indeed, there has been growing evidence that B. anthracis can directly infect non-phagocytic cells [32]. Therefore, our results suggest that B. anthracis–host interactions in the nasopharynx, and in particular with the NALT, are critical and should be the focus of future research.

The data obtained through gastric inoculation of spores reflect many of the characteristics of human gastrointestinal anthrax, including two forms of infection (oropharyngeal and lower gastrointestinal), intestinal abscesses, hemorrhage, necrosis, and epithelial barrier breakdown [2]. To our knowledge, this is the first establishment of a mouse model of gastrointestinal B. anthracis infection. This model should prove particularly useful in addressing questions regarding B. anthracis pathogenesis since mice offer a wide variety of immunologic and genetic tools that permit the determination of specific host factors involved in host–pathogen interactions. Furthermore, we observed that while abrasions to the mucosa were preferentially infected, gastrointestinal infection also occurred in the absence of mucosal damage, albeit with slower kinetics. Indeed, it has been hypothesized that the tendency of anthrax outbreaks to begin after drought conditions is due to abrasions in the mucosa caused by the consumption of sharp dried plants [11]. Similarly, preexisting lesions may predispose humans to infection [28,29]. The means by which B. anthracis invades the Peyer's patch in the absence of mucosal damage remains unknown, but may include uptake by M cells or dendritic cells [33].

In vivo bioluminescent imaging of B. anthracis has numerous possible applications. Some of these applications include real-time visualization of the effects of antibiotic therapy at different stages of B. anthracis infection, which is of particular interest because anthrax diagnosis often occurs at late stages of the disease, leading to ineffective treatment [11]. Another potential application is the analysis of different anti-anthrax vaccination strategies to determine where, when, and how efficiently the induced protective adaptive immunity terminates infection.

Taken together, the data presented in this manuscript suggest that the current paradigm of B. anthracis infection should be revisited. Unexpectedly, we found that B. anthracis spores germinate and establish infections at the initial site of inoculation in both inhalational and cutaneous infections, and that mice initially develop infections in Peyer's patches upon intragastric inoculation of spores. Since the elimination of toxin production does not affect virulence in the mouse model of infection [8,9], one would predict that our models are valid representations of infections with toxinogenic capsulated strains, though whether differences will be seen in the presence of both toxin production and capsule synthesis remains to be determined experimentally.

## Supporting Information

Figure S1 Histological Analysis of Bioluminescent Organs Show Infection, Inflammation, Necrosis, and Hemorrhaging Sections derived from intragastrically infected mice after 38 h (Peyer's patch, spleen, and lung) or intranasally infected mice after 48 h (mandibular lymph node). Left and center: hematoxylin and eosin (H&E) staining. The major observations were: in the Peyer's patch, enlargement, breakdown of the dome epithelial barrier, infiltration of numerous polymorphonuclear cells, and the presence of necrosis; in the infected lymph node, extensive necrosis; in the spleen, hemorrhaging and necrosis; in the infected lung, congestion, inflammation, and hemorrhaging. Right: Gram staining. Numerous Gram-positive bacteria are visible in all samples. Bacteria in the lung are restricted to lung tissues and are not found in the alveolar space.
(10 MB PDF)

### Accession Numbers

The National Center for Biotechnology Information (http://www.ncbi.nlm.nih.gov) accession numbers for the genes and gene products discussed in this paper for B. anthracis are protective antigen, *pagA* (AF268967); lethal factor, *lef* (M29081); Edema factor, *cya* (M24074); capsule synthesis operon, *capBCAD* (D85765), and *capE,* which can be found on the sequence of plasmid pX02 (AF188935). The number for the P. luminescens luciferase operon, *luxCDABE,* is M90093.

## Abbreviations

BHI: brain–heart infusion
BLI: bioluminescent imaging
CFU: colony-forming units
NALT: nasal-associated lymphoid tissue
