# Supplementary material for: Primary Involvement of Pharynx and Peyer's Patch in Inhalational and Intestinal Anthrax
Source: PLoS Pathog. 2007 Jun 1;3(6):e76. doi: 10.1371/journal.ppat.0030076 (PMC1885272; doi:10.1371/journal.ppat.0030076)

SUPPLEMENTARY FIGURE 1

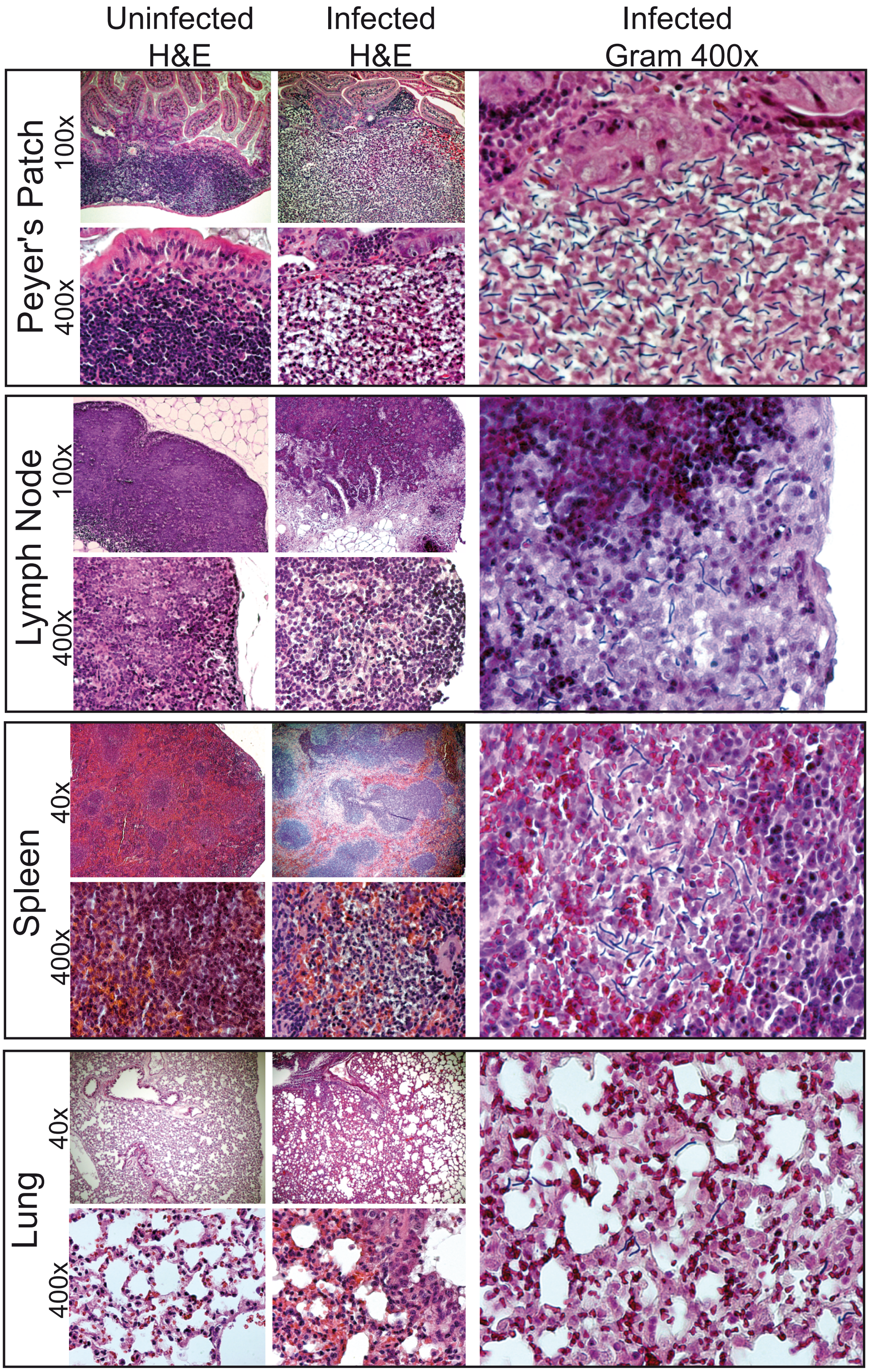

Supplement: Supplementary file 1 [file ppat.0030076.sg001.pdf]
